# Delirium in older patients undergoing aortic valve replacement: incidence, predictors, and cognitive prognosis

**DOI:** 10.1186/s12877-021-02100-5

**Published:** 2021-03-02

**Authors:** Marc Humbert, Christophe J. Büla, Olivier Muller, Hélène Krief, Pierre Monney

**Affiliations:** 1 Service of Geriatric Medicine and Geriatric Rehabilitation, Department of Medicine, University of Lausanne Medical Center (CHUV), Lausanne, Switzerland; 2 Service of Cardiology, Department of Cardio-Vascular Medicine and Surgery, University of Lausanne Medical Center (CHUV), Lausanne, Switzerland

**Keywords:** Aortic stenosis, Delirium, Transcatheter aortic valve replacement, Surgical aortic valve replacement, Society of thoracic surgeons risk score

## Abstract

**Background:**

Transcatheter aortic valve replacement is increasingly performed in frail older patients who were previously ineligible for a standard surgical procedure. The objectives of this study are to determine delirium incidence, predictors, and relationship with cognitive performance at 3-month follow-up in older patients undergoing aortic valve replacement (AVR).

**Methods:**

Patients (*N* = 93) aged 70 years and older, undergoing transcatheter (TAVR, *N* = 66) or surgical (SAVR, *N* = 27) aortic valve replacement in an academic medical center were enrolled in this prospective cohort study. Delirium was assessed using the Confusion Assessment Method (CAM) on postoperative days 1, 2, 3, and 7. Data on patients’ socio-demographics, functional status (including instrumental activities of daily living (IADL), and surgical risk scores (including Society of Thoracic Surgeons (STS) risk score), were collected at baseline. Cognitive status was assessed with the Mini-Mental Status Exam (MMSE) and the Clock Drawing Test (CDT) at baseline and 3 months after AVR.

**Results:**

Delirium occurred in 21 (23%) patients, within the first three postoperative days in 95% (20/21) of the cases. Delirium incidence was lower in TAVR (13/66 = 20%) than SAVR (8/27 = 30%) patients, but this difference was not statistically significant (*p* = .298). Patients with delirium had lower baseline cognitive performance (median MMSE score 27.0 ± 3.0 vs 28.0 ± 3.0, *p* = .029), lower performance in IADL (7.0 vs 8.0, *p* = .038), and higher STS risk scores (4.7 ± 2.7 vs 2.9 ± 2.3, *p* = .020). In multivariate analyses, patients with intermediate (score > 3 to ≤8) and high (score > 8) STS risk scores had 4.3 (95%CI 1.2–15.1, *p* = .025) and 16.5 (95%CI 2.0–138.2, *p* = .010), respectively, higher odds of incident delirium compared to patients with low (score ≤ 3) STS risk scores. At 3-month follow-up (*N* = 77), patients with delirium still had lower MMSE score (27.0 ± 8.0 vs 28.0 ± 2.0, *p* = .007) but this difference did not remain significant once adjusting for baseline MMSE (β-coefficient 1.11, 95%CI [− 3.03–0.80], *p* = .248).

**Conclusions:**

Delirium occurred in about one in five older patients undergoing AVR, almost essentially within the first three postoperative days. Beside cognitive performance, STS risk score could enhance the identification of high-risk older patients to better target preventative interventions.

**Supplementary Information:**

The online version contains supplementary material available at 10.1186/s12877-021-02100-5.

## Background

High-risk older patients who were formerly ineligible for surgical aortic-valve replacement (SAVR) can now be treated with transcatheter aortic valve replacement (TAVR), with better survival than conservative treatment up to 5 years after the procedure [1–4]. TAVR also proved non-inferior to SAVR in terms of survival [5–7] so that TAVR indication progressively broadened to patients with intermediate [8, 9] and even low surgical risk [10–13]. Aortic valve replacement is thus increasingly performed for severe stenosis and future increase up to 17′000 and 9′000 new TAVR candidates each year are expected in the European countries and the USA, respectively [14].

Delirium is a however a frequent complication among frail older patients undergoing cardiac surgery in general and in valve replacement in particular [15–18]. Indeed, several studies investigated the incidence of, risk factors for, and outcomes associated with postoperative delirium after aortic valve replacement. Results showed wide variations in incidence rates that ranged from as low as 0% up to 44.6% in a systematic review of TAVR [19] and up to 50.7 and 66% in studies of SAVR [18, 20]. These variations likely result from differences in study design (retrospective vs prospective), as well as methods and timing of delirium assessment (single vs repeated assessments).

Results from studies that investigated risk factors of delirium showed that baseline cognitive performance was consistently a major predictor [21–24]. In contrast, results for other characteristics such as age or surgical risk scores are far more heterogeneous [19]. For instance, whereas some studies found a significant association between Euroscore results and delirium risk after cardiac surgery, others did not [22, 25–27]. Indeed, determining whether surgical risk scores could also predict a patient’s risk of delirium, regardless of cognitive performance, could be very helpful in practice.

Data on cognitive outcome associated with delirium in patients undergoing aortic valve replacement are also less clear. A recent meta-analysis that focused on cognitive outcomes after TAVR showed no overall significant changes in cognition up to 34 months after the procedure [28]. In contrast, a meta-analysis on cognitive outcome after SAVR found an increased likelihood of cognitive decline after the procedure [29]. Other results from individual studies also appear conflicting, ranging from improved cognition in the immediate perioperative period after TAVR [28, 30], especially among patients with lower preoperative cognition [31–33], to transient perioperative worsening [34] or preservation of overall cognitive performances [35]. Overall, only few studies specifically looked at the potential role of delirium incidence on cognitive outcome and showed conflicting results. Eide et al. found that delirium had no impact on cognitive function at 1-month and 6-month follow-up [20], whereas Schoenenberg et al. observed that most subjects whose cognitive performance deteriorated at 6-month after TAVR experienced a delirium [31]. Clarifying the potential role of post-operative delirium in mediating cognitive outcome appears especially important to better target preventative interventions to high-risk older patients.

To get further insight on these issues, the present study aimed: 1) to determine the incidence of delirium in patients undergoing aortic valve replacement; 2) to identify predisposing factors for delirium; 3) to compare length of stay in patients with and without delirium; and 4) to investigate the association between delirium and cognitive performance at 3-month follow-up. The hypothesis was that patients with postoperative delirium will be more likely to decline in their cognitive performance from baseline to 3-month follow-up assessments.

## Methods

This is a prospective monocentric study in the University of Lausanne Medical Center (CHUV), a tertiary hospital in Lausanne, Switzerland. The study was conducted in the clinical and intermediate care units of the Service of Cardiology. At the time of the study no formal measures for delirium prevention were implemented but geriatric consult service for delirium management was available. The study was approved by the State Human Research Ethics Committee (Protocol 319/12). Participants were informed of the study goals and gave written consent.

### Patient selection

Eligible patients were those aged 70 years or older diagnosed with severe aortic valve stenosis (defined as an aortic valve area < 1 cm^2^ or < 0,6 cm^2^/m^2^) who underwent TAVR or SAVR between March 2014 and December 2017.

Patients were excluded if medically unstable, requiring an emergency intervention, suffering from concomitant severe aortic insufficiency and/or mitral valve disease requiring specific intervention. In addition, those with prior cardiac surgery (e.g coronary bypass, aortic valve replacement), endocarditis or constrictive pericarditis were also excluded.

### Data collection

For each patient, socio-demographic data (age, sex, living situation: living at home without formal help vs living at home with formal help vs living in a nursing home) were collected at baseline (Additional file 1). In addition, data on functional (Katz’s basic Activities of Daily Living – ADLs - [36] and Lawton’s instrumental ADLs [37]), cognitive (Mini Mental State Examination [38] and Rouleau’s Clock Drawing test [39]), and affective (miniGDS [40]) status as well as mobility performance (Tinetti’s Performance Oriented Mobility Assessment [41] and walking speed [42] were collected at baseline and at the 3-month follow up visit. Data on length of stay was collected from the hospital administrative database.

For every patient, alongside echocardiographic and coronary angiography data, the Society of Thoracic Surgery (STS) risk score [43] and Euroscore II [44] were systematically calculated. These scores gather information about a patient’s socio-demographic, biological, and cardio-vascular as well as other diseases status to predict her/his risk to develop postoperative complications.

#### Assessment of delirium

A trained research nurse assessed the patient before the intervention and each morning thereafter on postoperative days 1, 2, 3, and 7 for delirium using a validated French version [45] of the Confusion Assessment Method [46]. Each participant was attributed to one single research nurse who performed all the assessments in-person. Interactions with the nursing staff in charge of the patient and her/his relatives was not routinely performed but could occur in case of doubt about a recent change in cognition. Attention was assessed using simple commands and the month of the year backward.

### Statistical analysis

Simple descriptive statistics (percentage, median, interquartile range [IQR]) were used to determine delirium incidence. Predisposing factors for delirium were identified from bivariate comparisons in patients with and without delirium, using the Wilcoxon rank-sum test variables and Fisher exact test for continuous and categorical variables, respectively. Then, a multivariate regression analysis was performed with the occurrence of delirium as a dichotomous outcome and the type of AVR as well as baseline characteristics associated with delirium in bivariate analysis as candidate variables for adjustment.

Length of stay in patients with and without delirium were compared using Wilcoxon rank-sum test.

Finally, bivariate and multivariate analyses were performed to predict cognitive performance at 3-month follow-up, adjusting for baseline cognition, and the presence of delirium.

All analyses were performed using STATA program (version 14.2).

## Results

**Fig. 1:**
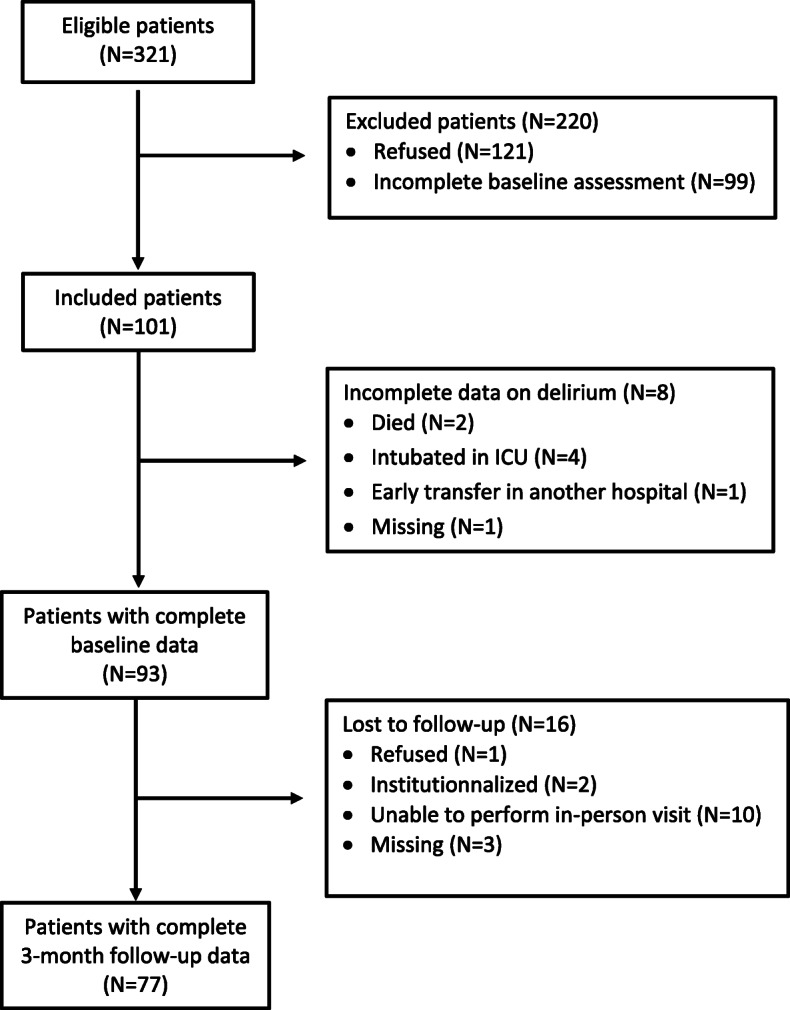
Study flow chart of patients’ enrolment and follow-up

From the original eligible population (*N* = 321), 121 refused to participate and 99 could not be assessed at baseline for logistical reasons leaving a total of 101 included patients (Fig. 1). In addition, 8 patients could not be assessed for postoperative delirium secondary to ICU admission with intubation (*N* = 4), early transfer to another hospital (*N* = 1), death (*N* = 2), and logistical problem (*N* = 1), leaving a final sample of 93 patients. Compared to the 93 remaining patients, those 8 patients had similar median age (82.0 [IQR 4.0] vs 82.1 [10.3] years, *p* = .725), baseline median MMSE (28.0 [3.0] vs 28.0 [3.0], *p* = .829) and IADLs (8.0 [0.5] vs 8.0 [2.0], *p* = .151) scores. They were more frequently men (75% vs 55%, *p* = .460), had higher STS risk score (4.4 [4.5] vs 3.4 [2.7], *p* = .174), and were less often treated with TAVR (38% vs 71%, *p* = .105), but none of these differences achieved statistical significance.

Baseline characteristics of included patients are presented in Table 1. Participants’ median age was 82.1 years, 45% were women, essentially living at home (99%), and only a quarter (24%) received formal in-home help. Overall, 66 (71%) of the patients underwent a TAVR and 27 (29%) underwent SAVR.

**Table 1:** Characteristics of the overall population at baseline and comparisons in subjects with and without post-operative delirium

Characteristics	Total population *N* = 93 (100%)	Post-operative delirium		*p*-value‡‡
		Yes *N* = 21 (22.6%)	No *N* = 72 (77.4%)	
***SOCIO-DEMOGRAPHIC***				
Age (years) Median / IQR	82.1 / 10.3	83.1 / 7.6	81.8 / 10.1	.038
Male sex (%)	51 (54.8)	13 (61.9)	38 (52.8)	.460
Living at home n(%)	91 (98.9)	21 (100.0)	70 (98.6)	1.000
Receiving formal inhome help n(%)	22 (23.9)	8 (38.1)	14 (19.7)	.083
***GERIATRIC ASSESSMENT***				
MMSE score * Median / IQR	28.0 / 3.0	27.0 / 3.0	28.0 / 3.0	.029
Clock Drawing Test † Median / IQR	10.0 / 2.0	9.0 / 2.0	10.0 / 2.0	.445
Depressive symptoms ‡ n(%)	23 (24.7)	4 (19.1)	19 (26.4)	.577
Basic ADLs § Median / IQR	6.0 / 0.0	6.0 / 0.0	6.0 / 0.0	.544
Instrumental ADLs ‖ Median / IQR	8.0 / 2.0	7.0 / 3.0	8.0 / 1.5	.038
Performance Oriented Mobility Assessment ¶ Median / IQR	28.0 / 5.0	27.0 / 5.0	28.0 / 4.0	.279
Gait speed # Median / IQR (m/s)	0.68 / 0.56	0.65 / 0.56	0.70 / 0.56	.443
***SURGICAL RISK SCORES***				
STS risk score **				
Median/IQR	3.4/2.7	4.7/2.7	2.9/2.3	.020
• High risk (score > 8), n(%) • Intermediate risk (score > 3 and ≤ 8),n(%) • Low risk (score ≤ 3), n(%)	9 (9.7) 40 (43.0) 44 (47.3)	4 (19.1) 12 (57.1) 5 (23.8)	5 (6.9) 28 (38.9) 39 (54.2)	.023
Euroscore 2 ††				
Median/ IQR High risk (score ≥ 15), n(%)	4.2 / 3.9 3 (3.2)	4.2 / 5.7 2 (9.5)	4.2 / 3.8 1 (1.4)	.308 .127

(*) *MMSE* Mini Mental Status Examination [38]; scores range from 0 to 30, with higher score indicating better cognitive performance

(†) *CDT*^:^ Clock Drawing Test [39]; scores range from 0 to 10 with higer score indicating higher cognitive performance

(‡) mini Geriatric Depression Scale [40]; scores range from 0 to 4 with significant depressive symptoms if score ≥ 1/4

(§) Basic ADLs: Basic Activities of Daily Living [36]: bathing, dressing, using the toilet, transferring between bed and chair, maintaining continence, and feeding; scores range from 0 to 6, with higher scores indicating higher function

(‖) Instrumental ADLs: Instrumental Activities of Daily Living [37]: using the phone, grocery shopping, cooking, housekeeping, doing the laundry, using transportation, taking medications, and handling finances; scores range from 0 to 8, with higher scores indicating higher function

(¶) Performance Oriented Mobility Assessment [41]: scores range from 0 to 28 with higher scores indicating better balance

(#) Gait speed: measured at usual pace over 6 m

(**) STS risk score = Society of Thoracic Surgeons risk score in cardiac surgery [43]:

“Low risk” if score ≤ 3; “Intermediate risk” if score > 3 and ≤ 8; “High risk” if score > 8

(††) Euroscore II = risk score in cardiac surgery [44]: indicates postoperative mortality rate

(‡‡) *P*-values from Wilcoxon rank-sum and Fischer exact test for continuous and categorical variables, respectively

### Incidence of postoperative delirium

Postoperative delirium occurred in 21 (23%) of the 93 patients. Most (18/21, 86%) developed delirium already on the first postoperative day, and all but one (20/21, 95%) within the first 3-day period after the procedure.

### Factors associated with postoperative delirium

Comparisons of baseline characteristics in patients with and without delirium are presented in Table 1. In bivariate analysis, patients with delirium had significantly lower cognitive performance (MMSE score [IQR] 27.0 [3.0] vs 28.0 [3.0], *p* = .029), lower performance in Instrumental ADLs (7.0 [3.0] vs 8.0 [1.5], *p* = .038), and higher STS risk score (4.7 [2.7] vs 2.9 [2.3], *p* = .020) than patients without delirium. Indeed, the proportion of patients who developed postoperative delirium steadily increased across levels of baseline STS risk score, from 11% in the lowest risk group (STS risk score ≤ 3), to 30% in the intermediate risk group (STS risk score > 3 to ≤8), and to 44% in the highest risk group (STS risk score > 8). Patients with TAVR had lower incidence of delirium (13/66 = 20%) than those with SAVR (8/27 = 30%), but this difference did not reach statistical significance (*p* = .298). 

In multivariate analysis (Additional file 1 Table B), a higher cognitive performance at baseline remained associated with significantly decreased odds of developing delirium (AdjOR 0.8, 95%CI 0.7–0.9, *p* = .001).

**Fig. 2:**
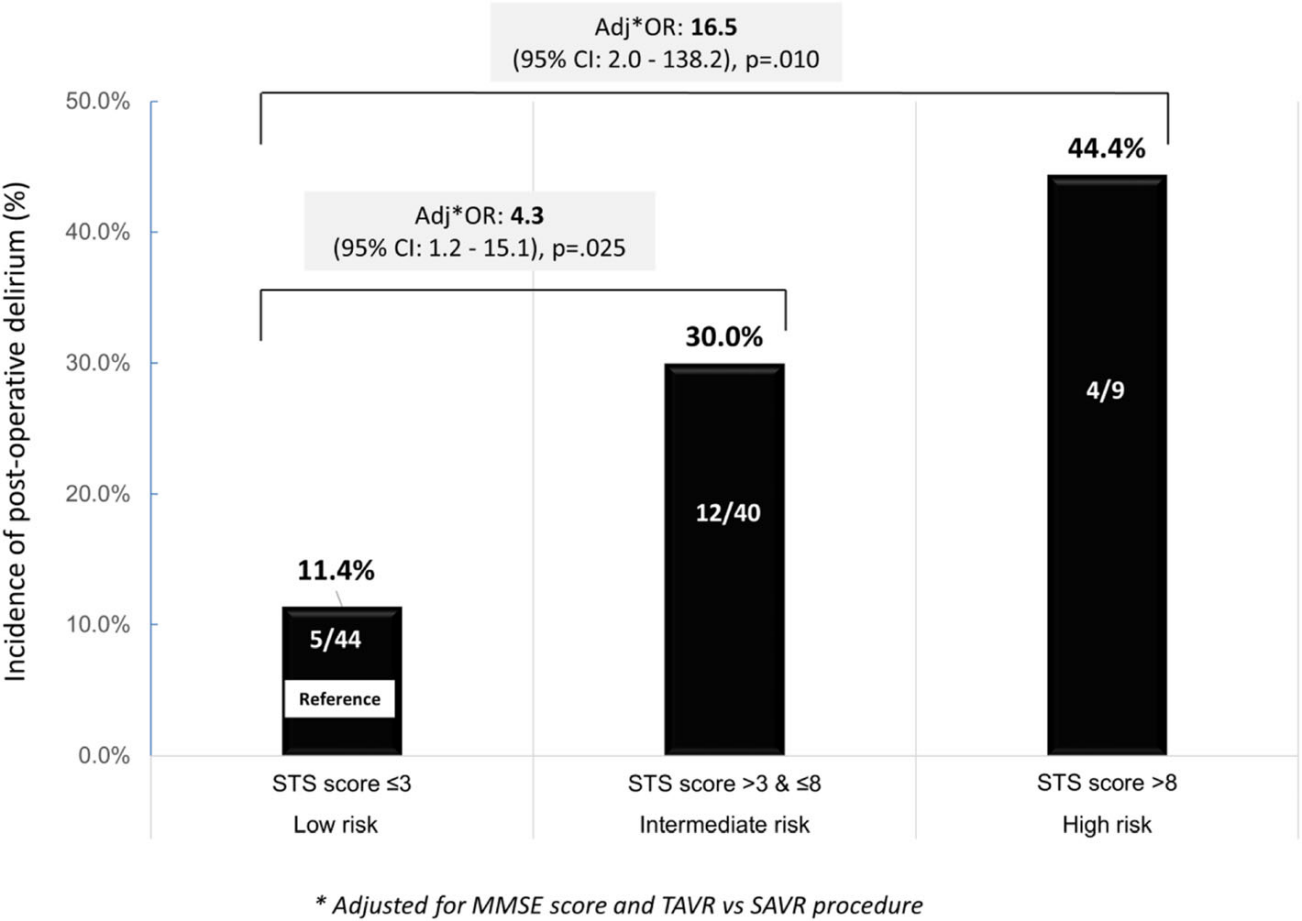
Incidence rate of delirium according to Surgical Thoracic Society score (STS risk score)

Similarly, an independent association between STS risk score and delirium remained significant as patients with intermediate (score > 3 to ≤8) and high (score > 8) STS risk scores had 4.3 (95%CI 1.2–15.1, *p* = .025) and 16.5 (95%CI 2.0–138.2, *p* = .010), respectively, higher odds of incident delirium compared to patients with low (score ≤ 3) STS risk score (Fig. 2). Finally, TAVR was associated with 80% (AdjOR 0.2, 95% CI 0.1–0.8, *p* = .020) lower odds of delirium than SAVR. In contrast, baseline performance in instrumental ADLs did not remain associated with delirium once adjusting for the other covariates. The final multivariate model correctly classified 80.7% of the patients with an area under the ROC curve of 0.80.

### Length of stay

Overall length of hospital stay was 11.2 (SD 6.9) days. Although patients with delirium had longer stays than patients without delirium (14.5 days; SD 11.4, IQR 7 vs 10.3 days; SD 4.6, IQR 7), this difference did not achieve statistical significance (*p* = .128 from Wilcoxon rank-sum test).

### Cognitive outcome at 3-month follow-up

Sixteen (17%) of the 93 patients initially enrolled did not complete the 3-month follow-up assessment because they were unable to travel to the examination site (*N* = 10), had been institutionalized (*N* = 2), refused (*N* = 1), or were lost (*N* = 3). Compared to those who did complete the 3-month follow-up, these patients were older (83.6 [12.9] vs 81.8 [9.9] years, *p* = .433), had lower baseline MMSE (27.5 [3.0] vs 28.0 [3.0], *p* = .257) and instrumental ADLs (6.5[3.0] vs 8.0 [2.0], *p* = .074) scores, and higher STS risk score (4.4 [4.9] vs 3.2 [2.4], *p* = .079). They also did more frequently experience a delirium (38% vs 20%, *p* = .117), but, due to the limited sample size, none of these differences achieved statistical significance.

Among patients who completed the 3-month follow-up (Table 2), those who experienced delirium had lower MMSE at follow-up (27.0 [8.0] vs 28 [2.0], *p* = .007). However, this association did not remain once adjusting for baseline MMSE performance (β coefficient -1.11, 95% CI [− 3.03–0.80], *p* = .248) (Additional file 1 Table C).

**Table 2:** Comparisons of cognitive and functional performance (in basic and instrumental activities of daily living ADLs) at 3-month in patients with and without post-operative delirium

Characteristics at 3-month follow-up	Total population (*N* = 77)	Post-operative delirium at baseline		*p*-value
		Yes (*N* = 15)	No (*N* = 62)	
MMSE ^a^				
Median / IQR Range (min, max)	28.0 / 3.0 17.0 (13.0, 30.0)	27.0 / 8.0 16.0 (13.0, 29.0)	28.0 / 2.0 9.0 (21.0, 30.0)	.007
Clock Drawing Test ^b^				
Median / IQR Range (min, max)	10.0 / 1.0 10.0 (0.0, 10.0)	9.0 / 3.0 5.0 (5.0, 10.0)	10.0 / 1.0 10.0 (0.0, 10.0)	.136
Basic ADLs ^c^				
Median / IQR Range (min, max)	6.0 / 0.0 3.0 (3.0, 6.0)	6.0 / 0.0 3.0 (3.0, 6.0)	6.0 / 0.0 2.0 (4.0, 6.0)	.678
Instrumental ADLs ^d^				
Median / IQR Range (min, max)	7.0 / 2.0 7.0 (1.0, 8.0)	7.0 / 2.0 7.0 (1.0, 8.0)	8.0 / 2.0 6.0 (2.0, 8.0)	.147

(^a^) *MMSE* Mini Mental Status Examination [38]; scores range from 0 to 30, with higher score indicating better cognitive performance

(^b^) Clock Drawing Test [39]; scores range from 0 to 10 with higer score indicating higher cognitive performance

(^c^) Basic ADLs: Basic Activities of Daily Living [36]: bathing, dressing, using the toilet, transferring between bed and chair, maintaining continence, and feeding. Scores range from 0 to 6, with higher scores indicating higher function

(^d^) Instrumental ADLs: Instrumental Activities of Daily Living [37]: using the phone, grocery shopping, cooking, housekeeping, doing the laundry, using transportation, taking medications, and handling finances. Scores range from 0 to 8, with higher scores indicating higher function

## Discussion

The present study shows that more than one out of five (23%) patients who underwent an AVR developed delirium in the postoperative period, a figure within the mid-range reported in previous studies [21–24, 47–49]. Overall, these results highlight the need to propose preventative interventions such as hydration, mobilization, reorientation, or prevention of constipation to at-risk patients.

In this regard, a contribution of the current study is to show that the STS risk score was a strong predictor of delirium, independent of a patient’s cognitive status and type of AVR. Indeed, the risk of delirium increased exponentially across level of STS risk, increasing 4- and 16-fold in patients with intermediate and high risk, respectively, compared to those with low STS risk score. Thus, even though this score was not intended to identify patients at risk for delirium, it gathers extended information about a patient health (hypertension, diabetes, chronic lung disease, renal failure) that likely also reflects his or her potential vulnerability to develop postoperative complications, including delirium. These results extends those of previous studies that showed a significant association between frailty and postoperative delirium in TAVI [22, 50] as well as other types of surgery [51–53]. Thus, STS risk score could certainly be considered as a proxy measure of frailty in older patients who are candidate for AVR. Future studies in larger population should provide more precise estimates of the “dose-response” relationship between STS risk score and delirium risk. Predicting the probability of delirium would nicely complete the list of adverse events currently provided when calculating the STS risk score (http://riskcalc.sts.org/stswebriskcalc/calculate, accessed June 26th, 2020).

Another interesting contribution of the present study is to show that, among older patients selected for AVR, delirium incidence was significantly lower in those selected to undergo TAVR rather than SAVR once adjusting for baseline cognition and STS risk score. This finding is even more striking when considering that all TAVR patients, at the time of the study, underwent general anesthesia, a possible additional risk factor for delirium [50, 54].

Results also strengthen previous evidence in showing that, among patients’ baseline characteristics, cognitive performance was a strong independent predictor of postoperative delirium. In contrast, performance in Instrumental ADL did not remain an independent predictor of delirium once adjusting for patients’ cognitive performance at baseline and the type of AVR.

The present study did not observe a significant association between delirium occurrence and cognitive performance at 3-month follow-up. Likely this observation results from the combined effect of a selective attrition of the frailest patients who more frequently had experienced a delirium, and the limited statistical power resulting from this attrition.

This study has several limitations such as its limited sample size and the attrition at follow-up that limited its ability to identify significant association. Another limitation is the exclusion of patients with more complex valve disease and medical instability. Thus, generalization of results to this type of patients should be very cautious. Finally, the methodology to assess delirium could also be criticized as it was based on the CAM and not a complete DSM-V-based evaluation. In addition, it was performed only once daily and was limited to postoperative days 1,2,3 and 7. This study has also several strengths, including the use of a large set of validated tools performed by a single assessor.

## Conclusions

About one in five senior patients who underwent AVR developed delirium after the procedure. Current results also extend previous information about patients most likely to develop delirium in showing that, besides cognitive status, the STS risk score could help to stratify delirium risk among these patients. Finally, even though results at follow-up were inconclusive, directions of the observed changes strongly suggest that patients who experienced delirium after AVR should be further assessed at distance to monitor their cognitive evolution.

## Supplementary Information

**Additional file 1.**

## Data Availability

Data supporting the results of the current study are available from the corresponding author upon request.

## Abbreviations

AdjOR: Adjusted odds ratio
ADL: Activities of daily living
AVR: Aortic valve replacement
CAM: Confusion assessment method
CDT: Clock drawing test
IADL: Instrumental activities of daily living
IQR: Interquartile range
miniGDS: Mini geriatric depression scale
MMSE: Mini-mental status exam
OR: Odds ratio
ROC: Receiver operating characteristic
SAVR: Surgical aortic valve replacement
STS: Society of thoracic surgeons
TAVR: Transcatheter aortic valve replacement
