# Supplementary material for: Delirium in older patients undergoing aortic valve replacement: incidence, predictors, and cognitive prognosis
Source: BMC Geriatr. 2021 Mar 2;21:153. doi: 10.1186/s12877-021-02100-5 (PMC7927377; doi:10.1186/s12877-021-02100-5)
Supplement: Supplementary file 1 — Additional file 1. [file 12877_2021_2100_MOESM1_ESM.docx]

**APPENDIX A**

Socio-demographic data collected for this study :

- Age
- Sex
- Living situation : living at home without formal help vs living at home with formal help vs living in a nursing home

**APPENDIX B**

**Table B**. Results from the multivariable logistic regression analysis predicting the incidence of post-operative delirium (N=93 participants).

| Characteristics | OR | 95% CI | p-value |
| --- | --- | --- | --- |
| ***Cognitive performance*** * | 0.8 | 0.7-0.9 | .001 |
| ***Surgical risk score by STS*** †   - - Intermediate (STS risk score >3 & ≤ 8)   - High (STS risk score >8)   *(ref is Low risk, STS score ≤3)* | 4.3  16.5 | 1.2-15.1  2.0-138.2 | .025  .010 |
| ***Type of operation*** ‡  TAVR  *(ref SAVR procedure)* | 0.2 | 0.1-0.8 | .020 |

OR: Odds ratio; 95% CI: 95% confidence intervals

(*) Using the Mini Mental Status Examination MMSE) score; scores range from 0 to 30, with higher score indicating better cognitive performance.

(†) STS risk score = Society of Thoracic Surgeons risk score in cardiac surgery :

"Low risk" if score ≤3; "Intermediate risk" if score >3 and ≤8; "High risk" if score >8

(‡) TAVR: Transthoracic aortic valve replacement; SAVR: Surgical aortic valve replacement

**APPENDIX C**

**Table C.** Results from the multivariable analysis predicting cognitive performance at the Mini Mental Status Exam (MMSE) in participants who completed the 3-month follow-up exam (N=77)

| Characteristics | Coefficient* | 95% CI | p-value |
| --- | --- | --- | --- |
| ***Post-operative delirium*** | -1.11 | -3.03-0.80 | .248 |
| ***Cognitive performance at baseline*** † | 0.51 | 0.29-0.74 | <.001 |

(*) Coefficient from multivariable linear regression analysis

(†) Cognitive performance at the Mini Mental Status Exam (MMSE) at initial assessment prior to intervention
